# Development of a Water-Soluble Nanomicellar Formulation Loaded with Trans-Resveratrol Using Polyethylene Glycol Monostearate for the Treatment of Intracerebral Hemorrhage

**DOI:** 10.3390/pharmaceutics16111462

**Published:** 2024-11-15

**Authors:** Kengo Banshoya, Aoi Machida, Saki Kawamura, Tetsuhiro Yamada, Riko Okada, Yui Kawamoto, Hikaru Kimura, Sachi Shibata, Yuhzo Hieda, Yoshiharu Kaneo, Tetsuro Tanaka, Masatoshi Ohnishi

**Affiliations:** 1 Faculty of Pharmacy and Pharmaceutical Sciences, Fukuyama University, Fukuyama 729-0292, Hiroshima, Japan; machida@fukuyama-u.ac.jp (A.M.); p7119028@fukuyama-u.ac.jp (S.K.); p7120097@fukuyama-u.ac.jp (T.Y.); p7120020@fukuyama-u.ac.jp (R.O.); p7120029@fukuyama-u.ac.jp (Y.K.); kimurahikaru@yamaguchi-u.ac.jp (H.K.); hieda@fukuyama-u.ac.jp (Y.H.); kaneoyoshiharu@gmail.com (Y.K.); tanaka@fukuyama-u.ac.jp (T.T.); 2 Graduate School of Pharmacy and Pharmaceutical Sciences, Fukuyama University, Fukuyama 729-0292, Hiroshima, Japan; 3 Pharmacy Department, Yamaguchi University Hospital, Ube 755-8505, Yamaguchi, Japan; 4 Faculty of Health and Welfare Science, Okayama Prefectural University, Soja 719-1197, Okayama, Japan; sachi_shibata@fhw.oka-pu.ac.jp

**Keywords:** trans-resveratrol, micellar formulation, water-soluble, polyethylene glycol monostearate, intracerebral hemorrhage

## Abstract

**Background/Objectives:** Trans-resveratrol (Res) has been reported to possess many biological activities, including neuroprotective effects, owing to its anti-inflammatory and antioxidant properties. However, Res has very low water solubility, which limits its therapeutic application. In this work, we formulated water-soluble micellar formulations incorporating Res using polyethylene glycol monostearate (stPEG). **Methods:** These formulations (stPEG/Res) were developed using five types of stPEG containing 10, 25, 40, 55 and 140 PEG repeat units. The formulations were characterized for Res content, water solubility, particle size, zeta potential, precipitation, biodistribution, and efficacy against neuronal and motor dysfunction in intracerebral hemorrhage (ICH). **Results:** Intravenous administration of stPEG40/Res, which demonstrated particle size, water solubility, and biodistribution properties suitable for intravenous administration, suppressed neurological and motor dysfunction following in a collagenase-induced ICH mouse model. These effects were inhibited by zinc protoporphyrin-9, an inhibitor of the antioxidant enzyme heme oxygenase-1, suggesting that Res contributes to antioxidant enzyme expression and anti-inflammatory activity. **Conclusions:** The stPEG/Res micellar formulation developed in this study may offer a promising therapeutic approach for ICH treatment.

## 1. Introduction

Intracerebral hemorrhage (ICH) is a neurological disease caused by nontraumatic bursting of blood vessels in the brain. It is the second most common stroke subtype after ischemic stroke [1]. The mortality rate within the first month after ICH onset exceeds 30%, with only 14% of survivors regaining functional independence after 12 months [2]. The primary treatment for ICH is surgical evacuation of the hematoma, which itself can cause secondary damage. Due to the lack of pharmacological treatment options, ICH remains the most challenging subtype of stroke [3]. Therefore, there is an urgent need to develop safe and effective treatment strategies. Numerous studies have demonstrated that factors such as oxidative stress caused by red blood cell lysis products and plasma components lead to brain damage after ICH [4]. Oxidative stress is involved in the pathophysiology of numerous brain diseases, including neurodegenerative diseases, depression, and ischemic stroke [5]. Importantly, oxidative stress is also a major mediator of secondary brain injury after ICH [5]. Thus, oxidative stress plays an important role in brain injury following ICH [6]. Due to the lack of effective pharmacological strategies for the treatment of ICH, researchers have increasingly turned to traditional and alternative medicines, particularly natural products [7].

One natural product that is an effective antioxidant for the treatment of ICH is trans-resveratrol (Res). Res is the most useful, common, and abundantly available polyphenol in pharmaceutical and dietary supplements [8]. Res is a safe compound that has been proven to exhibit many neuroprotective effects in various neurological diseases, such as stroke, Alzheimer’s disease, and Parkinson’s disease [9,10,11,12]. Importantly, frequent oral administration of Res has been shown to reduce brain injury in experimental ICH models [13]. However, when Res is administered orally, most of it is rapidly metabolized in the liver and excreted in urine and feces [14]. In addition, the need for pre-administration of Res and the fact that some patients with ICH are comatose makes oral administration of Res unsuitable for the treatment of ICH. Res is highly lipophilic, with a low water solubility of approximately 0.03 mg/mL [15]. Therefore, a study has been reported in which Res was dissolved in dimethyl sulfoxide and administered intravenously to investigate its therapeutic effect in ICH model animals [16]. However, intravenous administration of organic solvents, such as dimethyl sulfoxide, is clinically problematic due to their toxicity. Water-insoluble compounds are usually difficult to formulate as injectable drugs, and Res is no exception. As a solubilized formulation of Res that can be safely administered intravenously, there is a report on nano-formulation using lipids [17]. Lipid-based nano-formulations loaded with compounds other than Res have also been reported to be effective against ICH [18,19]. However, lipid-based nano-formulations are expensive and require complex manufacturing procedures. Therefore, a water-soluble Res injection formulation that overcomes these issues is required for the treatment of ICH.

One way to improve the performance of hydrophobic compounds is to use polymeric micelle systems [20,21,22,23]. Polymeric micelles, composed of amphiphilic polymers containing polyethylene glycol (PEG), have several advantages, including improved drug solubilization, stability, and ease of preparation and storage [24]. Polyethylene glycol monostearate (stPEG) is generally regarded as an essentially nontoxic and nonirritating material, and this amphiphilic block polymer containing PEG can be used in topical, oral, and intravenous formulations [25]. Furthermore, stPEG has various HLB values depending on the PEG length [25]. This makes it possible to use stPEG with optimal properties for solubilization of hydrophobic compounds.

We have successfully developed water-soluble stPEG micellar formulations of α-tocopherol and coenzyme Q10 [26,27]. Similar to these, Res is a poorly water-soluble antioxidant. In the present study, we developed novel intravenous Res micelle formulations using five different PEG lengths of stPEG, encapsulating Res in the inner hydrophobic part of the micelle. These micelles were investigated for their Res content, particle size, zeta potential, precipitation, saturation solubility, toxicity, and therapeutic effects in ICH models.

## 2. Materials and Methods

### 2.1. Materials

Res was purchased from Tokyo Chemical Industry (Tokyo, Japan). Medetomidine and pentobarbital were obtained from Kyoritsu Seiyaku (Tokyo, Japan). MuLV reverse transcriptase was purchased from Thermo Fisher Scientific (Waltham, MA, USA). Random primers were obtained from Takara Bio (Shiga, Japan). Other primers were obtained from Eurofins Genomics (Tokyo, Japan). The LightCycler FastStart DNA Master SYBER Green I was purchased from Roche Diagnostics Japan (Tokyo, Japan). Collagenase type IV was purchased from Sigma-Aldrich (Tokyo, Japan). The MTX-LDH was sourced from Kyokuto Seiyaku (Tokyo, Japan). Zinc protoporphyrin-9 (ZnPPIX) was purchased from Cayman Chemicals (Ann Arbor, MI, USA), and paraformaldehyde (PFA) was purchased from Nacalai Tesque (Kyoto, Japan). Mouse anti-neuronal nuclei (NeuN) antibody was purchased from Merck Millipore (Burlington, MA, USA). Biotinylated anti-mouse IgG (H + L) and an avidin-biotinylated horseradish peroxidase complex (Vectastain Elite ABC Kit) were obtained from Vector Laboratories (Burlingame, CA, USA). stPEG140 was kindly provided by Kao (Tokyo, Japan), and other stPEGs, reagents, and solvents were obtained from FUJIFILM Wako Pure Chemical (Osaka, Japan). All other chemicals and reagents used were of the highest grade commercially available and were used without further purification.

### 2.2. Cell Culture and Animals

Dulbecco’s Modified Eagle Medium (DMEM), penicillin/streptomycin, and trypsin/EDTA solution were purchased from FUJIFILM Wako Pure Chemical (Osaka, Japan). Fetal bovine serum (FBS) and fetal horse serum (HS) were purchased from Japan Bioserum (Fukuyama, Japan). PC-12 cells (established by Greene L.; JCRB0733), a rat pheochromocytoma cell line commonly used as a neuroblast model, were provided by JCRB Cell Bank (Osaka, Japan). The cells were maintained in DMEM supplemented with 5% FBS, 10% HS, 100 U/mL penicillin, and 100 μg/mL streptomycin. They were cultured at 37 °C in a humidified atmosphere containing 5% CO_2_. Four-week-old male ddY mice were purchased from Shimizu Laboratory Supplies (Kyoto, Japan) and housed in cages with sawdust bedding and free access to commercial food and distilled water.

### 2.3. Preparation of stPEG/Res

The stPEG/Res micelles were prepared by dissolving stPEG (400 mg) in 80 mL of distilled water under magnetic stirring. Subsequently, a solution of Res (40 mg) dissolved in 2 mL of acetone was slowly added to each stPEG solution using a syringe while stirring. These solutions were stirred for 3 h at room temperature to volatilize the acetone and then lyophilized with light shielding to obtain stPEG/Res micelle powder. Samples were stored at 4 °C, protected from light. Distilled water or 5% glucose was added to the micelle powder before use, followed by sonication.

### 2.4. High-Performance Liquid Chromatography (HPLC) Analysis

Res was quantified using HPLC. The HPLC system consisted of an LC-20AD pump (Shimadzu, Kyoto, Japan) and a variable-wavelength ultraviolet detector (SPD-20A, Shimadzu, Kyoto, Japan). The detection wavelength was set at 305 nm, and a 4.6 × 150 mm C18 reversed-phase column (TSKgel ODS 80TM, Tosoh, Tokyo, Japan) was maintained at 40 °C. The mobile phase comprised methanol, distilled water, and acetic acid (200:199:1, *v*/*v*/*v*), with a flow rate of 1.0 mL/min. The injection volume was 20 μL. The Res content (*w*/*w*)% of each micelle was calculated as follows:

Res content (*w*/*w*)% = (mass of Res loaded in micelles / mass of micelles) × 100

### 2.5. Measurement of Particle Size and Zeta Potential

The average particle size and zeta potential of each stPEG/Res micelle were measured using a Malvern Zetasizer Nano ZS (Malvern Instruments, Worcestershire, UK), as described previously [26]. Each micelle type was prepared at a concentration of 1 mg/mL.

### 2.6. Measurement of Saturation Solubility

The saturation solubility of each stPEG/Res sample was measured using the aforementioned HPLC system. Specifically, 50 mg of the Res-equivalent of stPEG/Res was mixed with 2 mL of distilled water and centrifuged at 10,000 rpm for 30 min. The supernatant was filtered through a 0.45 μm filter (Millex^®^-LH filter, Merck Millipore, Burlington, MA, USA) and diluted appropriately with the mobile phase for HPLC measurement.

### 2.7. Precipitation of stPEG/Res

The formation of stPEG/Res precipitates in the aqueous solution was evaluated. Briefly, 5 mg/mL solution of each stPEG/Res micelle formulation was prepared, filtered through a 0.45 μm filter, and stored in the dark at room temperature for 1 week. The formation of precipitates was visually confirmed.

### 2.8. In Vitro and in Vivo Toxicity

In vitro toxicity was evaluated by measuring the cytotoxicity of PC-12 cells using the lactate dehydrogenase (LDH) assay. Cells were seeded in 96-well plastic plates (CORNING, New York, NY, USA) at a density of 5 × 10^5^ cells/well. After 24 h of culture, the cells were treated with the test sample for 24 h. Subsequently, 25 µL of culture supernatant was transferred to each well of a 96-well plate pre-loaded with 75 µL/well of the chromogenic substrate for MTX-LDH assay. The plates were then incubated in the dark for 15 min at room temperature. The reaction was terminated with 1 M HCl, and the absorbance at 540 nm was measured using a microplate reader (Bio-Rad, Hercules, CA, USA).

The in vivo toxicity and the LD_50_ of stPEG40/Res were determined by limiting studies in five male ddY mice (4 weeks old). The mice received intravenous injections of 100 mg/kg Res-equivalent stPEG40/Res via the tail vein every 24 h. Mice that became moribund were euthanized for humane reasons (none of them exhibited morbidity in this study). Surviving animals were monitored for delayed mortality over a 14-day period.

### 2.9. RNA Preparation and Quantitative Real-Time PCR

Changes in the expression of antioxidant genes were measured in PC-12 cells. The cells were seeded at a density of 1 × 10^6^ cells/well in 35 mm dishes and treated with the test samples. After incubation for 6 h, the cells were washed with phosphate-buffered saline (PBS), and total RNA was extracted using the acid guanidinium thiocyanate-phenol-chloroform method. Complementary DNA (cDNA) was synthesized using MuLV reverse transcriptase and 50 pmol of random primers. Real-time reverse transcriptase-polymerase chain reaction (RT-PCR) was performed using a LightCycler 2.0 system (Roche Diagnostics Japan, Tokyo, Japan). In brief, reverse-transcribed cDNA, equivalent to 0.1 µg of total RNA, was used per reaction with LightCycler FastStart DNA Master SYBER Green I and 1 µM of primers. For the heme oxygenase-1 (HO-1) gene, the forward primer was 5′-TCTATCGTGCTCGCATG-3′ and the reverse primer was 5′-TTCCTCTGTCAGCAGTG-3′. For the glyceraldehyde 3-phosphate dehydrogenase (GAPDH) gene, the forward primer was 5′-TCTTCACCACCATGGAGA-3′ and the reverse primer was 5′-TGTCATGGATGACCTTGG-3′. Relative expression was calculated using the ΔΔCt method, with normalized to GAPDH as the endogenous control.

### 2.10. In Vivo ICH Mouse Model and Administration of stPEG/Res

To evaluate the therapeutic effects of stPEG/Res, a collagenase-induced ICH mouse model [28] was established. Mice were placed in a stereotactic frame (Narishige Scientific Instrument Lab, Tokyo, Japan) after an intraperitoneal injection (i.p.) of a mixture of medetomidine (0.3 mg/kg) and pentobarbital (40 mg/kg). The scalp was incised and the skull drilled. Collagenase type IV (0.03 U; volume: 3 µL/mouse; rate: 1 µL/min) or an equal volume of saline was injected into the right striatum (3.5 mm ventral from the skull surface, 2.2 mm lateral to the bregma midline suture, and 0.2 mm anterior to the coronal plane) using a Hamilton syringe (outer diameter: 0.019 inches; Hamilton, Reno, NV, USA). The Hamilton syringe was left for 5 min after injection and then gradually withdrawn. The scalp incision was closed with sutures and the mice were placed in a cage with ad libitum access to water and food. One hour prior to the collagenase injection, ZnPPIX, an inhibitor of HO-1 activity, was administered intraperitoneally at a dose of 5 mg/kg. ZnPPIX was dissolved in 0.1 M NaOH, neutralized with an equal volume of 0.1 M HCl, diluted 25-fold with 0.9% NaCl to a concentration of 1 mg/mL, and administered to the mice. Twenty minutes after the collagenase injection, stPEG40/Res (20 mg/kg Res-equivalent) or stPEG40 was administered via the tail vein. Both stPEG40/Res and stPEG40 were dissolved in 5% glucose before being administered.

### 2.11. Immunohistochemical Examinations

After 24 h of ICH induction, mice were reanesthetized with a mixture of medetomidine/pentobarbital i.p., and transcardial perfusion was performed with 10 mL of cold PBS and 4% PFA. Whole brains were excised, fixed in 4% PFA for 1 h, and then immersed in 15% sucrose overnight at 4 °C. The cerebellum was removed from the whole brain, frozen on dry ice, and stored at −80 °C until sectioning. Subsequently, 12 µm thick sections were prepared using a cryostat (Leica Biosystems, Wetzlar, Germany) and mounted on CREST-coated glass slides (Matsunami Glass Ind., Ltd., Osaka, Japan). NeuN antigen revival was performed by autoclaving the sections in 10 mM citric acid (pH 6.0) at 121 °C for 15 min (HIRAYAMA Manufacturing, Saitama, Japan). Sections were incubated with mouse anti-NeuN antibody (1:200) overnight at 4 °C following immersion in PBS containing 5% HS and 0.1% Triton X-100 for 1 h at room temperature. After washing with PBS, sections were incubated with biotinylated anti-mouse IgG (H + L) (1:200) for 1 h at room temperature. Finally, the sections were treated with an avidin-biotinylated horseradish peroxidase complex, and peroxidase activity was visualized using diaminobenzidine and H_2_O_2_.

Bright-field images were captured randomly using an inverted microscope (ECLIPSE TE300; Nikon, Tokyo, Japan) with a camera (DS-Ri2; Nikon). The NIS-Elements D software 4.30 (Nikon) was used for image acquisition and processing. Hematoma size was measured using coronal section images, including a syringe mask for collagenase injection. The area inside the hematoma was quantified using the ImageJ software 1.53t [29].

### 2.12. Behavioral Tests

The motor function of mice one day after ICH induction was assessed using corner-turn [30], modified beam-walking [29], and pole tests [31]. All animals were pretested, and investigators were blinded to group allocation.

In the beam-walking test, a 90 cm long beam, 1.5 cm wide and elevated 50 cm, was placed with dark home cages containing food on either end. The mice were placed at the center of the beam, and their behavior was observed and scored according to the criteria listed in Supplementary Table S2. Three trials of 2 min each were conducted, and the total score from all trials was calculated.

In the corner-turn test, the mice were directed toward a 30° corner, with the choice to turn left or right based on whisker contact with the wall. Ten trials were performed, and the percentage of turns to the right was calculated.

The pole test used a horizontal, rough-surfaced pole (8 mm diameter). The mice were placed on this pole with their forelimbs. Their behavior was observed and scored according to the criteria shown in Supplementary Table S3. Three trials of 30 s each were conducted, and the total score was calculated from all trials.

### 2.13. Statistical Analysis

Data are presented as mean ± standard deviation or standard error of the mean. ANOVA followed by Tukey’s multiple comparison test was used to analyze statistical differences between multiple groups. A *p*-value < 0.05 was considered statistically significant. R version 4.3.0 (Vienna, Austria) was used for all statistical analyses.

## 3. Results and Discussion

### 3.1. Characterization of stPEG/Res

stPEG forms nanoparticles at a concentration of approximately 1–10 µg/mL in an aqueous environment due to aggregation of stearyl groups [27]. At concentrations well above the CMC of stPEG, most stPEG molecules are thought to form micelles in water. Therefore, to efficiently incorporate Res into the hydrophobic core of the micelles, we used an stPEG aqueous solution at 5 mg/mL in the formulation, significantly exceeding the CMC. The Res content in the stPEG/Res formulation was determined using HPLC and ranged from 8.1% to 10.3% *w*/*w* (Table 1), which was approximately consistent with the initial weight ratio of Res during preparation. Table 1 summarizes the results of the DLS analysis and zeta potential measurements. DLS analysis of each formulation revealed that the diameter of stPEG10/Res was 85.4 nm, while the other stPEG/Res formulations had diameters of approximately 10–20 nm. The zeta potential values of all formulations were approximately −20 mV. Nanoparticles with diameters smaller than 100 nm are known to increase blood circulation to avoid rapid trapping by the reticuloendothelial system in the liver, spleen, and lungs [32]. The size of stPEG/Res was within this range, suggesting that it was suitable for intravenous formulations. The particle size and zeta potential of the PEGylated nanoparticles depend on the surface density, length, and structure of the PEG chains. The areal density of PEG on nanoparticles can affect its conformation, with a high surface density resulting in a brush-like appearance and a low surface density producing a mushroom-like appearance [33]. Owing to these factors, stPEG10/Res may have a larger particle size than the other stPEG/Res formulations.

Saturation solubility measurements revealed that the Res formulation with stPEG10 and 140 had low saturation solubility, less than 1 mg/mL Res equivalent, while the formulations consisting of stPEG25, 40, and 55 demonstrated very high saturation solubility, approximately 12 mg/mL Res equivalent (Table 1). The emulsifying and dispersing abilities of a nonionic surfactant can be estimated by calculating the hydrophile–lipophile balance (HLB) value from its chemical structure. The HLB values of stPEG10–140 were calculated using the formula proposed by Griffin [34]: HLB = PEG (wt. %)/5. The calculations resulted in HLB values of 12.2, 15.9, 17.2, 17.9, and 19.1 for stPEG 10–140, respectively. Griffin classified surfactants in the HLB range of 10–13 as o/w emulsifiers, 13–15 as detergents, and 15–18 as solubilizers [34]. The saturation solubility results suggest that a solubilizers with HLB values of 15–18 are suitable for solubilizing Res using nonionic surfactant stPEG.

**Figure 1:**
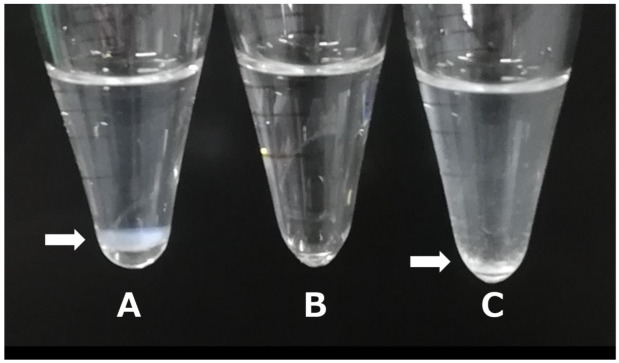
Precipitation of stPEG25/Res (**A**), stPEG40/Res (**B**), and stPEG55/Res (**C**) at a formulation concentration of 5 mg/mL in water after 1 week.

We further evaluated the precipitation properties of the three stPEG/Res samples with high saturation solubility after one week of storage. At a micellar concentration of 5 mg/mL, the stPEG40/Res solution remained clear, whereas the stPEG25/Res and stPEG55/Res solutions appeared cloudy (Figure 1). In addition, stPEG10/Res and stPEG140/Res at 5 mg/mL could not be dissolved without sonication. However, stPEG25-55/Res remained soluble without sonication under gentle agitation. It was suggested that stPEG10/Res and stPEG140/Res are unstable particles that tend to form aggregates and do not disperse unless energy such as sonication is applied.

### 3.2. Neuroprotective Effect of stPEG/Res in ICH

**Figure 2:**
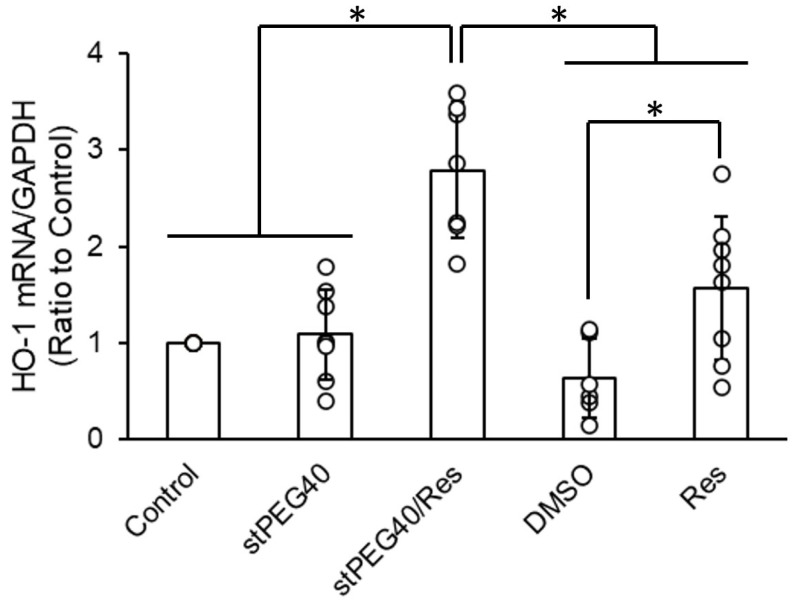
Effect of treatment with stPEG40/Res on the mRNA expression of HO-1 in PC-12 cells (*n* = 6–8). The values are presented as mean ± standard deviation. *—*p* < 0.05.

**Figure 3:**
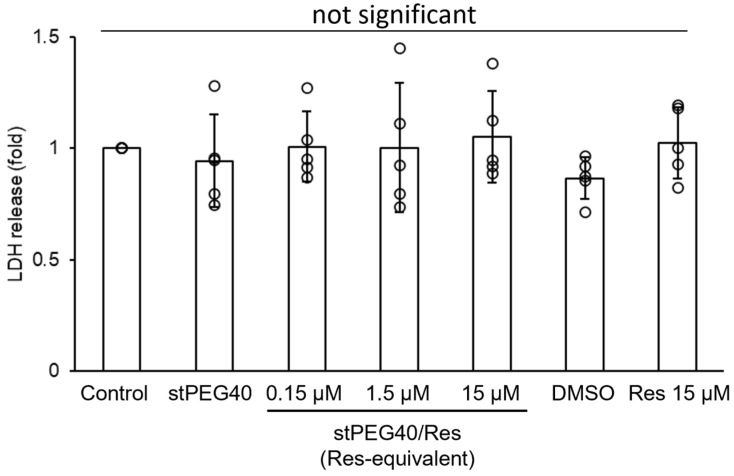
Cell injury in PC-12 cells induced by Res and stPEG40/Res, as assessed by the LDH assay. Cells were exposed to the samples for 24 h (*n* = 5). The values are presented as mean ± standard deviation.

Res exhibits protective activity against central nervous system damage by increasing the expression of antioxidant enzymes, including HO-1 [35]. Therefore, we focused on stPEG40/Res, which demonstrated no precipitation (Figure 1) and had the highest amount of Res concentration in the brain 10 min after administration (Supplementary Table S1). We inferred HO-1-mediated neuroprotective activity by measuring the increase in HO-1 gene expression following stPEG40/Res exposure in PC-12 cells (Figure 2). Exposure of 15 µM Res, a non-toxic concentration (Figure 3), significantly increased HO-1 mRNA expression compared to the control group in PC-12 cells. Furthermore, exposure of stPEG40/Res at an equivalent Res concentration of 15 µM significantly increased HO-1 mRNA expression compared to the control and the Res-exposed groups. The results showed that the Res formulation with stPEG may have a higher drug efficacy. stPEG promotes the intracellular translocation of loaded drugs [36]. The greater increase in HO-1 gene expression after exposure to this formulation may have been due to the enhanced internalization of Res by stPEG.

Following administration of stPEG40/Res at 20 mg/kg Res-equivalent dose, the average Res concentration in the brain was 0.75 µg/brain (Supplementary Table S1), approximately 11 µM. Given the results shown in Figure 2 and Figure 3, this concentration was expected to increase HO-1 expression with low toxicity. Therefore, we evaluated the in vivo toxicity of stPEG40/Res and the in vivo efficacy of stPEG40/Res following ICH. To evaluate the in vivo toxicity of stPEG40/Res, we performed LD_50_ measurements, which indicated that the LD_50_ for stPEG40/Res was higher than that of the 100 mg/kg Res-equivalent (Table 2).

**Figure 4:**
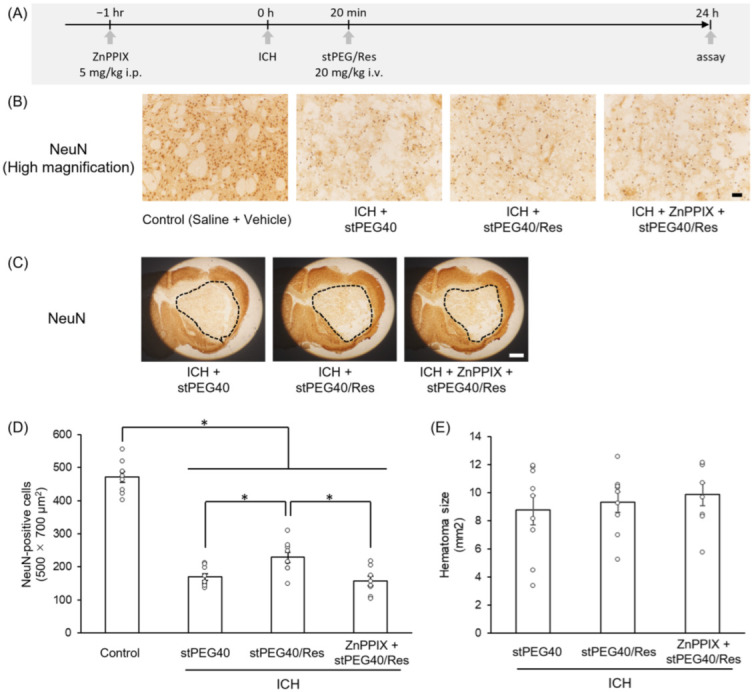
Neuroprotective effect of stPEG40/Res against intracerebral hemorrhage (ICH). (**A**) Experimental schedule using ICH model. (**B**) Magnified images of NeuN-immunopositive signals in the perihematomal area 24 h after ICH. Scale bar = 50 mm. (**C**) Representative images of NeuN immunostaining in the hematoma 24 h after ICH. Scale bar = 1 mm. Weak signals inside the dotted line are considered as a hematoma. (**D**) Number of NeuN-positive cells in the periphery of the hematoma (*n* = 8–9). (**E**) Summary of the hematoma size (*n* = 8–9). The values are presented as mean ± standard error. *—*p* < 0.05.

Based on previous results, we evaluated the efficacy of stPEG40/Res at a 20 mg/kg Res-equivalent dose in an ICH model (Figure 4A), which was sufficiently lower than the LD_50_ value and was expected to achieve pharmacologically effective Res concentrations in the brain. The in vivo efficacy of stPEG/Res against ICH was evaluated using a collagenase-induced ICH mouse model, a widely used animal experimental model [37]. The neuroprotective effects of stPEG40/Res were assessed by measuring nerve damage and hematoma size through immunohistochemical staining with an anti-NeuN antibody. The hematoma size 24 h after ICH induction was not significantly different between the solvent and stPEG40/Res groups (Figure 4E). In contrast, the number of NeuN-positive neurons around the hematoma was significantly higher in the stPEG40/Res group than that in the solvent group (Figure 4B–D). Furthermore, administration of the HO-1 inhibitor ZnPPIX prior to stPEG40/Res treatment reversed the increase in NeuN-positive neurons caused by stPEG40/Res (Figure 4B–D). Thus, the neuroprotective effects of stPEG40/Res were mediated by HO-1.

**Figure 5:**
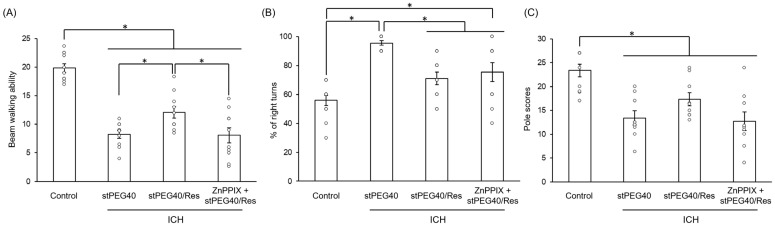
Efficacy of stPEG40/Res in alleviating motor dysfunction caused by intracerebral hemorrhage (ICH). (**A**) Effects of ICH and stPEG40/Res on beam walking ability (*n* = 10–11). (**B**) Effects of ICH and stPEG40/Res on corner turn test scores (*n* = 11–12). (**C**) Effects of ICH and stPEG40/Res on pole test scores (*n* = 9–10). The values are presented as mean ± standard error. *—*p* < 0.05.

Given the neuroprotective effects of stPEG40/Res, we expected that this formulation would be effective against motor dysfunction caused by ICH. We investigated its effect in suppressing motor dysfunction (Figure 5). Intravenous administration of stPEG40/Res at 20 mg/kg Res-equivalent to an ICH mouse model significantly improved performance on the beam walking test and corner turn test compared to the vehicle-administered group (Figure 5A,B). In addition, pole test scores tended to improve with stPEG40/Res administration (Figure 5C). Similar to the immunohistochemical staining evaluation, we performed a previous study in which ZnPPIX was administered. Regarding the beam-walking and pole test scores, the groups administered with both ZnPPIX and stPEG40/Res showed scores comparable to the vehicle-administered group (Figure 5A,C). In other words, similarly to the examination of the neuroprotective effect, ZnPPIX inactivated the effect of stPEG40/Res in improving motor dysfunction. These results indicate that the neuroprotective and motor dysfunction-improving effects of stPEG40/Res are mediated via HO-1.

Based on these findings, we successfully developed stPEG/Res, a water-soluble Res formulation that is effective for treating ICH and can be easily administered intravenously, even to unconscious patients. Furthermore, Res has been reported to be effective against various diseases, such as cardiovascular disease [38], kidney disease [39], malignant tumors [40], and neurological diseases [9,10,11,12]. Thus, stPEG/Res may have potential applications as therapeutic agents for these diseases. In this study, we used ZnPPIX to show that the neuroprotective effects and motor dysfunction improvement effects of Res in ICH were mostly mediated through HO-1. To the best of our knowledge, this is the first study to show that the mechanism of action of Res in ICH is primarily mediated through HO-1. This research is an example showing that poorly soluble compounds that could not previously be used as therapeutic agents because of bioavailability issues can be used as therapeutic agents by incorporating them into nanoparticles such as nanomicelles using stPEG or other materials. We hope that this research will have an impact on the development of new therapeutic agents that solubilize poorly soluble compounds.

## 4. Conclusions

In this study, we developed a novel intravenous micelle formulation composed of Res and stPEG, which exhibited suitable properties for intravenous administration. This formulation is inexpensive and easy to prepare compared with other nano-formulations. Furthermore, in vitro experiments using PC-12 cells demonstrated that stPEG40/Res induced the gene expression of enzymes that protect against oxidative stress and inflammation. In vivo experiments using a collagenase-induced ICH mouse model showed that intravenous administration of stPEG40/Res provided neuroprotective effects and suppressed motor dysfunction following ICH. These effects may be due to the upregulation of antioxidant and anti-inflammatory enzyme expression by Res. Consequently, these results suggest that stPEG/Res represents a novel and effective therapeutic formulation for ICH treatment.

## Figures and Tables

**Table 1:** Properties of the stPEG/Res.

Sample	Res Content (% w/w)	Diameter (nm)	Zeta Potential (mV)	Saturation Solubility (mg/mL) †
stPEG10/Res	10.3 ± 1.8	85.4 ± 7.2	−20.7 ± 0.5	0.93 ± 0.03
stPEG25/Res	8.8 ± 0.2	10.6 ± 0.6	−16.0 ± 4.2	12.42 ± 0.24
stPEG40/Res	8.6 ± 0.6	11.8 ± 0.2	−17.6 ± 4.6	12.30 ± 0.35
stPEG55/Res	9.8 ± 0.6	11.4 ± 0.3	−18.9 ± 2.0	12.19 ± 0.48
stPEG140/Res	8.1 ± 1.2	16.6 ± 1.2	−21.3 ± 1.4	0.43 ± 0.01

Values are expressed as mean ± standard deviation (*n* = 3). † The saturation solubility immediately after dissolution is expressed as Res-equivalent.

**Table 2:** In vivo acute toxicity of stPEG40/Res in ddY mice.

Sample	LD_50_ (mg/kg)	95% Confidence Interval (mg/kg)
stPEG40/Res	>100 †	–

†—Res-equivalent.

## Data Availability

The original contributions presented in the study are included in the article/Supplementary Materials; further inquiries can be directed to the corresponding author.

## Supplementary Materials

The following supporting information can be downloaded at: https://www.mdpi.com/article/10.3390/pharmaceutics16111462/s1, File S1: Biodistribution to the brain. Table S1: Distribution of Res in the brain; File S2: Scoring criteria for behavioral tests. Table S2: Score criteria for modified beam-walking test; Table S3: Score criteria for pole test.
