# Supplementary material for: Development of a Water-Soluble Nanomicellar Formulation Loaded with Trans-Resveratrol Using Polyethylene Glycol Monostearate for the Treatment of Intracerebral Hemorrhage
Source: Pharmaceutics. 2024 Nov 15;16(11):1462. doi: 10.3390/pharmaceutics16111462 (PMC11597214; doi:10.3390/pharmaceutics16111462)
Supplement: Supplementary file 1 [file pharmaceutics-16-01462-s001.zip › pharmaceutics-3301248-supplementary.pdf]

Supplementary Materials

Development of a water-soluble nanomicellar formulation loaded with trans-resveratrol using polyethylene glycol monostearate for the treatment of intracerebral hemorrhage

Kengo Banshoya[1,#,*], Aoi Machida[1,2,#], Saki Kawamura[1], Tetsuhiro Yamada[1], Riko Okada[1], Yui Kawamoto[1], Hikaru Kimura[1,3], Sachi Shibata[4], Yuhzo Hieda[1], Yoshiharu Kaneo[1], Tetsuro Tanaka[1], Masatoshi Ohnishi[1,2,*]

1 Faculty of Pharmacy and Pharmaceutical Sciences, Fukuyama University, Gakuen-cho 1, Fukuyama, Hiroshima 729-0292, Japan

2 Graduate School of Pharmacy and Pharmaceutical Sciences, Fukuyama University, Fukuyama, Hiro-shima 729- 0292, Japan

3 Pharmacy Department, Yamaguchi University Hospital, Gakuen-cho 1, Ube, Yamaguchi 755-8505, Japan

4 Faculty of Health and Welfare Science, Okayama Prefectural University, 111 Kuboki, Soja, Okayama 719-1197, Japan

# These two authors contributed equally to this work

* Correspondence: kban@fukuyama-u.ac.jp (K. Banshoya), ohnishi@fukuyama-u.ac.jp (M. Ohnishi), +81-84-936-2111

File S1

Biodistribution to the brain

Biodistribution measurements were performed as previously described, with some modifications [1]. Four-week-old male ddY mice were injected via the tail vein with stPEG/Res at a dose of 20 mg/kg Res-equivalent in 200 µL of 5% glucose. After anesthetized, the mice were sacrificed, and 10 min post-administration, their brains were excised and weighed. The harvested organs were homogenized in methanol and left on ice for 1 h. The samples were then centrifuged at 15000 rpm for 20 min at 4°C, and the supernatants were collected. Each supernatant was passed through a 0.45-µm filter to prepare HPLC samples, which were then injected into the HPLC system.

HPLC was performed using an LC-40D pump (Shimadzu, Kyoto, Japan) and a variable-wavelength ultraviolet detector (SPD-40, Shimadzu, Kyoto, Japan). The detection wavelength was set to 305 nm, and a 4.6 × 150 mm C18 reversed-phase column (L-column2 ODS, Chemicals Evaluation and Research Institute, Tokyo, Japan) was maintained at 40°C. The mobile phase consisted of methanol, distilled water, and acetic acid (80:119:1, v/v/v) at a flow rate of 1.0 mL/min. The injection volume was 40 µL.

Supplementary Table S1. Distribution of Res in the brain. n = 4.

Sample	Res distribution (µg/brain)
stPEG25/Res	not detected
stPEG40/Res	0.75 ± 0.22
stPEG55/Res	not detected

[1] K. Banshoya, T. Nakamura, T. Tanaka, Y. Kaneo, Development of α-tocopherol nanomicellar formulation using polyethylene glycol monostearate for the oxidative stress-related disease, J. Drug Deliv. Sci. Technol. 61 (2021) 102310. doi:10.1016/j.jddst.2020.102310.

File S2

Scoring criteria for behavioral tests

Supplementary Table S2. Score criteria for modified beam-walking test.

score	criteria
0	Mouse falls immediately or does not move at all
1	Mouse falls within 2 min
2	Over 50% of mouse body is located under beam multiple times
3	Over 50% of mouse body is located under beam once
4	Hindlimbs slip multiple times
5	Hindlimbs slip once
6	Mouse holds for 2 min or crosses beam
-1	Mouse does not move more than 50% in 2 min

Supplementary Table S3. Score criteria for pole test.

score	criteria
1	Mouse grabs the bottom of the horizontal pole for 1-10 s and then falls
2	Mouse grabs the bottom of the horizontal pole for 11-20 s and then falls
3	Mouse grabs the bottom of the horizontal pole for 21-30 s and then falls
4	Mouse grabs the bottom of the horizontal pole for 30 s, but the left hindlimb and forelimb are hanging down the pole
5	Mouse grabs the bottom of the horizontal pole for 30 s, but the left hindlimb or forelimb is hanging down the pole
6	Mouse firmly grabs the bottom of the horizontal pole for 30 s but cannot sit on it
7	Mouse sits on the pole, but the left hindlimb and forelimb are hanging down
8	Mouse sits on the pole, but the left hindlimb or forelimb is hanging down
9	Mouse moves freely on the pole, and all limbs are stable
